# Living evidence syntheses for long COVID therapeutics: combining rigorous protocols to build efficiency while maintaining rigour

**DOI:** 10.1186/s13643-026-03178-x

**Published:** 2026-04-02

**Authors:** Tiffany Atkins, Paul Glasziou, Samantha Chakraborty, Tari Turner, Gabriel Rada, Oyungerel Byambasuren

**Affiliations:** 1 Institute for Evidence-Based Healthcare, Bond University, Gold Coast, QLD Australia; 2 Australian Living Evidence Collaboration, School of Public Health and Preventive Medicine, Monash University, Melbourne, VIC 3004 Australia; 3 Epistemonikos Foundation, Santiago, Chile; 4 Universidad del Desarrollo, Santiago, Chile

**Keywords:** Living systematic review, Methods, Therapeutics, Long COVID, Post-acute sequelae of COVID-19, COVID-19, SARSCoV2

## Abstract

**Background:**

Given the rapidly changing evidence, the creation and maintenance of a living systematic review database of therapeutics for long COVID is an ideal and necessary approach considering the rapidly changing evidence that continues to be identified. This paper describes methods and results of a collaboration between three teams who produced a living literature review on long COVID therapeutics—Australian Living Evidence Collaboration (ALEC), Bond University, and Epistemonikos COVID-19 L.OVE (Living Overview of Evidence) database.

**Methods:**

We took a collaborative and iterative approach to analyse the commonalities and differences between each project and develop an agreed comprehensive, collective approach. A plan for ongoing (monthly) updates and dissemination was built.

**Results:**

Despite minor differences, there was also a clear alignment of goals between the three teams. Differences in search strategy, search methods and screening criteria were identified, investigated, and resolved. A comparison of overlaps helped establish a common collaborative approach. A combined library of 218 randomised controlled trials and 56 systematic reviews was created which led to the optimised search strategy. The combined 218 RCT library covered 20 different treatment categories of which 14 were pharmacological and 6 were non-pharmacological. Further refinements and collaborations led to a transformed initial database library of 102 randomised controlled trials as of June 2024 before the team commenced monthly updates.

**Conclusions:**

Despite initial differences, a comprehensive search strategy based on the collaboration of the three teams was developed. Ongoing monthly updates were initiated and are now planned for well into the future to make continual and rapid updates to the library of evidence surrounding therapeutics for long COVID. Where global public health is concerned, it is valuable to review and refine processes in the early stages, so that they can be reliable. We recommend open collaboration to achieve the goal of creating accessible, efficient, and reliable evidence syntheses.

**Supplementary Information:**

The online version contains supplementary material available at 10.1186/s13643-026-03178-x.

## Introduction

Long COVID, also referred to as post-acute sequelae of COVID (PASC) or post-COVID condition [1] is a multi-symptom, complex health condition that can arise after SARS-CoV-2 infection. It is characterised by signs and symptoms that develop during or after an infection consistent with COVID-19, continue for more than 12 weeks and are not explained by an alternative diagnosis [2]. Long COVID usually presents as clusters of symptoms (most commonly including fatigue, neurological concerns, respiratory issues, and cardiovascular complications) that can affect any system in the body. These symptoms are often overlapping and can fluctuate and change over time [2]. The prevalence of long COVID, following acute COVID-19 or asymptomatic SARS-CoV-2 infection, is estimated at between 5 and 30%; leading to estimates that 65 million people globally are currently living with long COVID [1].

Effective treatments for long COVID remain unknown. Numerous research studies and evidence reviews have been published in the past 5 years, and over 100 are currently listed on the international trial registry ClinicalTrials.gov as ongoing [3]. However, research studies are heterogenous, and results are largely inconclusive [4–6]. Long COVID treatment remains an ongoing priority for healthcare systems internationally. This lack of a substantive body of evidence of effective treatments, and the expectation that further research will be rapidly published in the coming months and years, allows a living evidence synthesis approach to be used to identify potentially effective treatments.

This paper describes the collaboration between three teams who have come together to produce a living evidence synthesis on long COVID therapeutics—Australian Living Evidence Collaboration (ALEC), Bond University, and Epistemonikos COVID-19 L.OVE (Living Overview of Evidence) database. Prior to commencing the collaboration, each team was developing its own approach to generating a living synthesis for long COVID.

ALEC developed Australia’s national living clinical practice guidelines for COVID-19 and long COVID from 2020 to 2023; and were subsequently supported by the Australian Government 2023 Medical Research Future Fund PASC grant to develop and maintain an updated review of the evidence on long COVID to inform a future adaptive platform trial. The Bond University team obtained funding through the same initiative to develop a plan for a national adaptive platform trial for testing potential treatment interventions in Australia; this project included producing an up-to-date review of therapeutics for long COVID. Epistemonikos’ COVID-19 L.OVE is the largest open access database of evidence relevant for COVID‑19. It was established in 2020 and provides an organised, continually updated and easily accessible database of research studies on COVID-19, including long COVID.

The collaboration between the three groups was stimulated by a desire to create efficiencies across these projects and to share the results of the living evidence review on therapeutics for long COVID in an open access platform, to be used by evidence users globally. We had two objectives: to create a unified search for identifying all published trials on therapeutics for long COVID, and to develop a plan for the ongoing up-to-date dissemination of review findings.

## Methods

We took a collaborative and iterative approach to describe the commonalities between each project, discuss differences, and develop an agreed plan for continual updating.

### Step 1: considerations for initiating the collaboration

All three groups were focused on producing a living evidence review on therapeutics for long COVID. Each group intended to include systematic reviews (SRs) or randomised controlled trials (RCTs) for interventions to improve long COVID symptoms.

### Step 2: understanding differences in process and methodology across projects

Prior to establishing the collaboration, each group had already developed their search methods and started running regular searches. To inform a joint approach to the search methods, we compared the processes and search results of each team as of June 2024. In the first instance, we compared the eligibility criteria used by each team.

To compare the results of the three searches, we created a collated list of identified articles and then deduplicated the three pairs of results (Bond–ALEC, Bond–Epistemonikos, Epistemonikos–ALEC) to identify the common and unique studies. We discussed whether differences across the three groups with respect to eligibility criteria and search methods were likely to have accounted for the variations in the results of each search.

### Step 3: determining publication timeframes and methods

Publication timeframes were already aligned (with Bond and ALEC both planning to conduct monthly searches). These timelines aligned with methods that Epistemonikos already used, whereby COVID-19 L.OVE searches are updated daily or weekly depending on the source, and studies are screened on a monthly basis.

The Epistemonikos COVID-19 L.OVE platform is an open access database that was being used by the Epistemonikos team to disseminate updates of living evidence on therapeutics for long COVID. To enable wide access to the combined findings from the three searches, the teams discussed ways to make the living library accessible to the public with the aid of the Epistemonikos platform.

## Results

### Clear alignment of goals

Across the groups there was a clear alignment of project goals, where each team intended to produce monthly updates of new RCTs on interventions for long COVID. Table 1 summarises the main differences with respect to long COVID definition, decision rules for conducting a systematic review, and plans for outputs.

**Table 1:** Summary outline of the three collaborative teams and their initial differences

	Definition of long COVID used	Decision rules to conduct a systematic review	Plan for outputs
ALEC team	Our definition is based on the WHO definition “continuation or development of new symptoms 3 months after the initial SARS-CoV-2 infection, with these symptoms lasting for at least 2 months with no other explanation.”	Not specified	Monthly report to CIs, Open Science Platform
Bond team	Had a history of COVID-19 and persisting long COVID symptoms	When there are at least 3 trials on the same interventions, we check for registered trials and consider conducting a new SR	Open Science Platform, Open access publications
Epistemonikos	A range of physical and psychological symptoms and health issues that persist or develop after the acute phase of COVID-19, typically lasting for weeks, months, or even longer	Not specified	Not specified

### Differences in search strategy, search methods, and screening were identified

Each team used different search methods and search strategies, as outlined below.

#### Bond team search

The Bond team initially included 57 RCTs from a SR published by the Health Information and Quality Authority (HIQA) in Ireland [7]. Bond conducted a forward and backward citation analysis of all 57 included studies from this SR using the SpiderCite tool [8] on 23 May 2024 to search for additional relevant SRs and RCTs. Next, the Bond team performed a backwards citation analysis of all relevant SRs that were found in the above search on 28 May 2024 to screen for further relevant RCTs and SRs, also using the SpiderCite tool. Furthermore, the Bond team searched PubMed on 3 June 2024 for any additional RCTs using a simple search that combined terms for long COVID and treatment, restricted to randomised trials published from 2020 onwards (see Table 2 below).

**Table 2:** Bond simple PubMed search terms

We utilised the combined search terms of ‘long COVID’ AND ‘treatment’. We additionally used a “randomised controlled trial” filter combined with a date filter of “2020 onwards” to search for any additional RCTs.	

#### ALEC team search

In June 2024, building on the search methods for the COVID-19 living guidelines, the ALEC team established their search for long COVID that involved searching PubMed for randomised trials (no date restriction) and systematic reviews (published from January 2024).

This was supplemented by the EPPI-Centre’s quarterly scopes for long COVID literature [9]. The ongoing surveillance search was scheduled to run monthly in PubMed (see Table 3 below).

**Table 3:** ALEC Search terms

PubMed search for randomised trials((Post-Acute COVID-19 Syndrome[mh] OR "long covid*"[ti] OR "post covid*"[ti] OR "after covid*"[ti] OR "following covid*"[ti] OR ("post acute"[ti] AND (covid*[ti] OR sars*[ti]))) AND (Clinical Trial[pt] OR trial[ti] OR randomi*[tiab] OR randomly[tiab] OR placebo[tiab])) NOT (Systematic[sb] OR meta-analysis[pt] OR meta-analysis[ti] OR vaccine*[ti] OR vaccination*[ti])PubMed search for systematic reviews((Post-Acute COVID-19 Syndrome[mh] OR "long covid*"[ti] OR "post covid*"[ti] OR "after covid*"[ti] OR "following covid*"[ti] OR ("post acute"[ti] AND (covid*[ti] OR sars*[ti]))) AND (Systematic Review[pt] OR systematic[ti] OR Meta-Analysis[pt] OR meta-analysis[ti] OR cochrane database syst rev[ta])) NOT (vaccine*[ti] OR vaccination*[ti]) AND (2024:3000[pdat])	

#### Epistemonikos team search

Epistemonikos created and maintained the Living Overview of Evidence for COVID-19 (COVID-19 L.OVE https://app.iloveevidence.com/covid-19) [10] platform. This evidence is based on daily or weekly searches in 40 electronic databases, preprint servers and trial registries, and other sources like the studies included in COVID-19 SRs and manual searches. The articles are classified using a combination of automated and manual approaches into the different categories of population (e.g. people with post-COVID-19 conditions), interventions (e.g. any intervention, pharmacological interventions), study design, and whether the study is “reported” (published) or “not reporting” (unpublished) status. To identify the trials and reviews from this database, the results were filtered by “Prevention or treatment”, “people with post-COVID-19 conditions”, “Post-COVID-19 conditions” and by “reported data” only which was conducted on 2 July 2024 (SR search) and 9 July 2024 (RCT search).

### The identification of randomised controlled trials from each team

The Bond team identified 3186 articles from the backwards citation search of new SRs. Of these, 62 studies were full-text screened and only 50 RCTs were included from this source. Next, a simple PubMed search retrieved 314 records of which 18 were retrieved for full-text screening which led to a total of 13 RCTs being included from this database search. The Bond team also searched for additional RCTs from the 2338 studies identified from the forwards and backwards citation analysis of the initial 57 included RCTs from the HIQA SR [7]. This led to 51 full texts assessed which excluded 14 studies producing a total contribution of 37 RCTs from this source. Only 55 out of the 57 original RCTs from the HIQA SR were included as 2 were excluded due to study design. Overall, the Bond team identified a total of 155 RCTs from four different sources (backward citation search of SRs (*n* = 50), PubMed (*n *= 13), forwards and backwards search of RCTs from HIQA SR (*n* = 37), and original RCTs from HIQA SR (*n* = 55)).

The ALEC team retrieved a total of 399 records from PubMed. Of these, 257 records were excluded at the title and abstract screening stage, producing a total of 112 records from this team.

The Epistemonikos COVID-19 L.OVE platform identified a total of 94 studies [10]. Six studies were excluded, leaving 88 eligible for inclusion from this team.

**Fig. 1:**
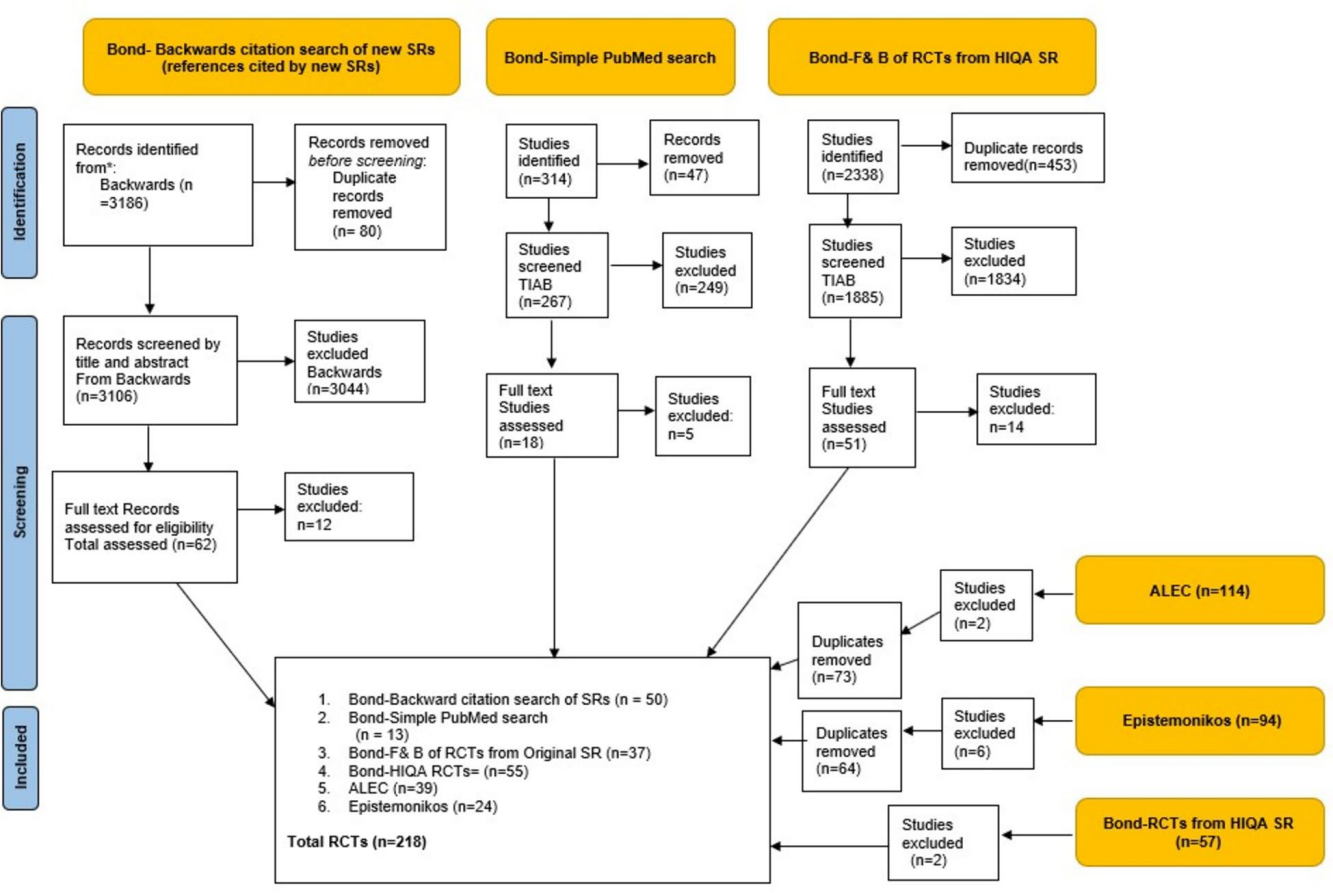
PRISMA flow diagram for the identification of RCTs

Overall, Bond identified 155 studies, ALEC 112, and Epistemonikos 88 before deduplicating across teams. The PRISMA flow diagram for the identification of RCTs is given within Fig. 1.

### The identification of systematic reviews from each team

**Fig. 2:**
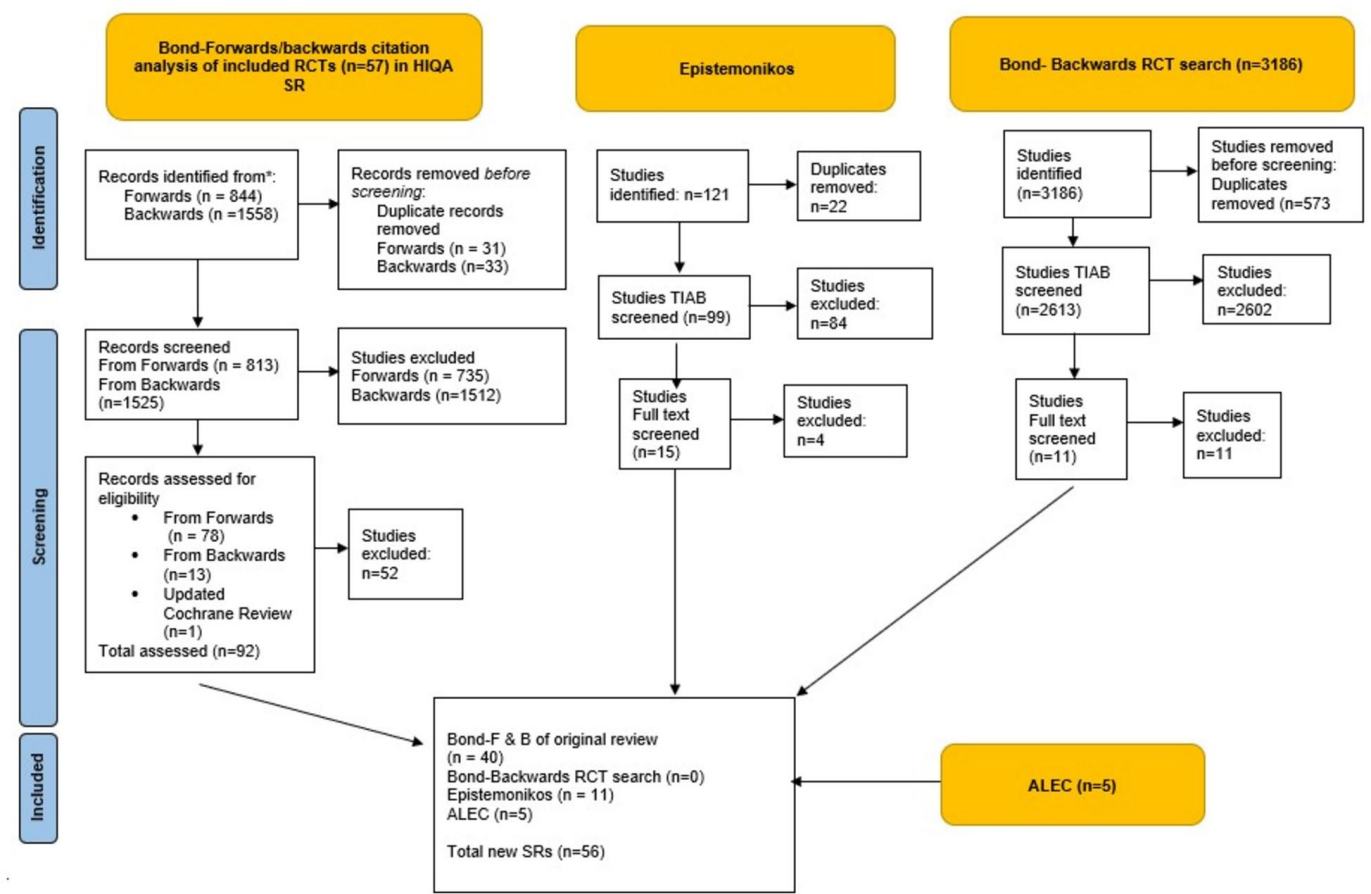
PRISMA flow diagram for the identification of SRs

Similar approaches to identifying RCTs by each team were applied to identifying SRs. Overall, Bond identified 40 SRs, Epistemonikos identified 11, and ALEC identified 15 (see Fig. 2 PRISMA flow diagram for the identification of SRs).

### Combining our results

We used the Bond team’s search results as the foundation to deduplicate their total initial library of 155 RCTs against each of the other team’s libraries. The ALEC team had an initial total library of 112 RCTs and after deduplication against what the Bond team had, 73 duplicates were removed and 39 RCTs were added to the overall library. Furthermore, Epistemonikos had an initial library of 88 studies and when deduplicated against Bond’s and ALEC’s library combined, 64 duplicates were removed, and 24 RCTs were added to the overall library.

The Bond team also deduplicated their original 51 SRs against 15 additional SRs from ALEC. This contributed an additional 5 studies to the SR library. Epistemonikos had an initial library of 121 SRs, of which 11 contributed to the total library (which was already included by Bond).

**Fig. 3:**
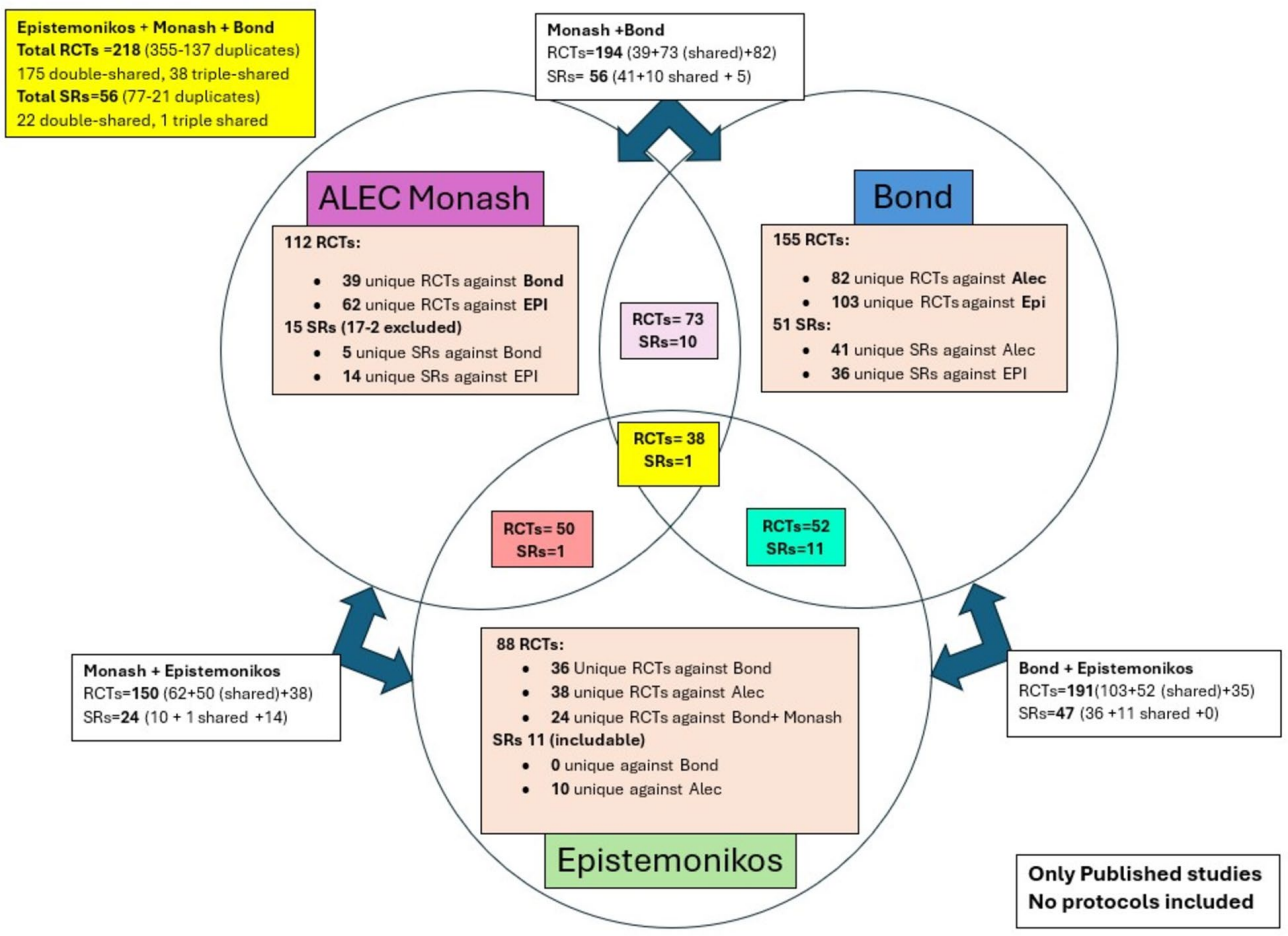
Venn diagram displaying the collaboration across the 3 teams as of June 2024. (Note that the number of studies reflects title and abstract screening for ALEC and full-text screening for the other teams)

Overall, a total of 218 RCTs and a total of 56 SRs were included within our combined library. Figure 3 below displays the collaboration across the three teams which shows that only 38 out of the 218 RCTs (17.4%) were found by all three teams. See also PRISMA flow diagrams for the identification of RCTs and SRs (Figs. 1 and 2).

### Differences in search strategy, search methods, and screening criteria were resolved

When we compared the inclusion and exclusion parameters of each search, we found that the Bond team had a much wider definition of long COVID compared to Epistemonikos and ALEC (Table 1). Upon discussion, we agreed to use the definition used by the ALEC team, as this closely aligns with international definitions of long COVID. When we applied the agreed definition to the 218 randomised trials initially identified, 116 were deemed not to meet the inclusion criteria, which left the starting database library with 102 RCTs. Table 4 below outlines how many RCTs each search team found out of the total initial library (*n* = 102) as well as how many were only found by that particular search team, and how many were found by all three teams.

**Table 4:** Number of RCTs each team found of the initial library (*n* = 102) (total, unique, and by all teams)

Search	Total RCTs found from search *n* (%)	# RCTs found only from that search (unique) (%)	# RCTs found by all 3 teams (%)
Bond searches:			
Bond- RCTs from HIQA SR	29 (28.4)	7 (6.9)	14 (13.7)
Bond-backwards search of new SRs	23 (22.5)	9 (8.8)	2 (2.0)
Bond-simple PubMed search	7 (6.9)	0 (0.0)	7 (6.9)
Bond-F and B of RCTs from HIQA SR	16 (15.7)	7 (6.9)	2 (2.0)
1. Total Bond	**75 (73.5)**	**23 (22.5)**	**25 (24.5)**
2. ALEC	**69 (67.6)**	**15 (14.7)**	**25 (24.5)**
3. Epistemonikos	**44 (43.1)**	**4 (3.9)**	**25 (24.5)**

Table 4 above reveals that the simple PubMed search by Bond did not uniquely contribute any RCTs to the library, whereas their backwards search of new SRs uniquely contributed the most (9 RCTs) compared to their other strategies. In total Bond found 23 unique RCTs from the initial library that were only found from the Bond searches. Furthermore, Bond found the highest percentage (75/102, 73.5%) of RCTs compared to the other teams. The ALEC search found the second highest percentage at 69/102 (67.6%), which found 15 unique RCTs. Epistemonikos found 43% of the RCTs from their searches (44/102), with 4 of these being unique to only the Epistemonikos search. Surprisingly, only 25 RCTs out of the 102 initial library (24.5%) were found by all three teams.

When we compared the three search methods, we found that the Bond team initially used a variety of methods to produce their library such as forwards/backwards of RCTs (without ever using specific search terms across databases). ALEC used a specific search strategy as did Epistemonikos. Bond developed a search strategy using search terms designed to search PubMed, Cochrane (including Central), and Embase (via Elsevier) aided by using their included studies that were retrieved from their search. Following the collaboration, the search strategy that was originally developed by Bond was tested to see if it returned most of the acquired articles including those from the other two teams (initial library) and 91% (93/102) of these were in fact found using the search strategy. The first monthly update utilised the existing ALEC’s search terms but then the Bond team’s search strategy was utilised thereafter. Further refinements of the search strategy may still be undertaken in the future if seen to be required or appropriate. However, the current search strategy (see Additional file 1) originating from Bond produces the ongoing monthly updates.

### Plan for ongoing update and dissemination

The Epistemonikos L.OVE platform had devised a taxonomy for therapeutic interventions for long COVID. Updates to the living database of studies are tracked in an Excel spreadsheet where monthly updates are added under these same taxonomy categories of therapeutic interventions. We reflected on this taxonomy and made some minor adjustments based on needs which included adding an “other” non-drug category and adding an “other”, “multiple”, and “olfactory/anosmia” pharmacological intervention categories. The original combined library and the refined library with taxonomy categories are outlined below in Table 5. The combined collaborative library of *n* = 218 RCTs covered 20 different treatment categories of which there were 14 pharmacological and 6 non-pharmacological categories. Whereas the refined initial library of *n* = 102 RCTs was spread across only 11 different categories (6 pharmacological and 5 non-pharmacological).

**Table 5:** Collaborative library (*n* = 218) and initial database library (*n* = 102) by taxonomy categories

Taxonomy categories	Number of SRs	RCTs in initial collaborative library (*n* = 218)	RCTs in initial library after collaborative rescreening (*n* = 102)
Pharmacological interventions			
Acetylcholinesterase inhibitor		1	0
Antidepressant		3	2
Antifibrotic		3	1
Antihistamine		1	0
Antivirals	1	3	0
Beta blockers		2	1
Corticosteroids	5	1	0
Enzyme Therapeutics		1	0
Mood stabilizer		1	0
Targeted drugs		2	2
Multiple	2	2	0
NSAIDs (*anti-inflammatory)		1	0
Olfactory function/anosmia		22	11
Other (BrainMax, AXA1125)		3	2
Non-pharmacological			
Physical activity and physical therapy	31	67	37
Therapeutic procedures	6	33	17
Complementary and alternative medicine	5	20	6
Behavioural, psychological, educational	2	12	6
Diet and dietary supplements	2	33	17
Other non-drug		7	0
Number of studies	**56**	**218**	**102**

After each monthly search update, we will send any new RCTs found to Epistemonikos COVID-19 L.OVE platform where they will add in any RCTs that are not currently present in their database. This way the public will have access to the most up-to-date library for the treatments of long COVID. Please see Additional file 2 for the full list of the initial 102 RCT references within the library (as of June 2024) prior to the start of the monthly updates.

## Discussion

To develop a comprehensive database of controlled trials that assessed treatments for long COVID, we merged three independently developed databases. While there was moderate overlap between pairs of databases, less than 20% of all trials before full-text (*n* = 218) and only 25% after full-text (*n* = 102) were identified by all three databases. All three databases had differences in scope as well as search strategies which could explain the modest overlap. Both the differences in search methods and differences in scope warranted discussions to agree the scope and methods for a common comprehensive database of trials which could be maintained.

Our case study illustrates the complexity in trying to develop and maintain a database of all trials in a specific condition. In particular, it highlights the need to use multiple techniques and approaches in trying to maintain a database of trials in a specific clinical topic. For example, the Bond University team focused initially on a seed set of articles (RCTs from one SR), and an iterative “spidering” (forward and backward citation searching) to identify additional articles. Some of these additional articles were likely to fall outside the scope of a standard search strategy. Hence, the combining search approaches of the three groups is more likely to achieve a comprehensive database. Even so, the modest overlaps suggest some additional studies might be missed by all three approaches. Hence individual systematic review using the combined database may sometimes also require supplementary methods.

Our case study also illustrates the importance of individual investment of time and resources to identify differences and develop suitable solutions. Although the broad scope of the review was aligned across the groups, individual differences in search methods, screening criteria, and technology to conduct the literature review warranted flexibility and transparency amongst the teams. Additionally, each team’s motivation to create efficiencies for users of the review by combining our reviews into a single trustworthy review was crucial for enabling the success of this collaboration. These elements of a successful living evidence collaboration align international efforts to reduce duplication and increase access to high-quality evidence syntheses internationally [11, 12].

Several single-topic trial databases have been developed previously. Notably, the Cochrane Schizophrenia Group has for decades maintained a comprehensive database of all trials of treatment in schizophrenia [13] an approach adopted by some other Cochrane groups such as the tobacco addiction and renal. To facilitate speedy systematic reviews on demand, the Cochrane Schizophrenia Group also completes a risk of bias assessment and data extraction on all identified trials [14]. This speed of reviews was a major driver for our long COVID database because of the need to inform treatment arms of a long COVID platform trial and for policy making. We currently produce updates of the trials database each month—effectively a living scoping review.

In addition to identifying all current trials, such a database has two other needs. First, a taxonomy of possible interventions, that is mutually exclusive and exhaustive—allowing the arms from each new trial to be categorised. While relatively straightforward for drug interventions, this is much more challenging for the non-drug interventions, which are often complex and/or poorly described. Second, in addition to published trials, the database should comprehensively identify preprints and registered studies; the database then should be study focused rather than publication focused. Both challenges need to be recognised as critical parts of the process.

We believe that developing condition-based databases will be a useful process in selected areas. This is particularly for emerging conditions such as SARS-CoV-2 and long COVID, but also for areas where there is a frequent policy or clinical need for reviews. Though worthwhile to allow an ongoing living scoping process and rapid systematic reviews, developing such a database faces several challenges which will be aided by the multiple methods illustrated in this case study.

## Conclusion

Initially, there were minor differences in methods across each of the three teams, but there also continued to be a clear alignment of project goals. A collaborative review approach was able to be established. From this, a comprehensive refined search strategy was developed. Ongoing monthly updates were initiated and are planned for well into the future using this comprehensive yet collaborative search strategy to make continual updates to the database library of evidence surrounding therapeutics for long COVID. This continually evolving database library will inform treatment arms of a long COVID platform trial, and for policy making; thereby, researchers will have the most up-to-date information to inform treatments amongst the rapidly incoming evidence. This same methodological process might be useful when applied in any other areas of evidence-based research that are predicted to quickly grow or change.

## Supplementary Information

Additional file 1. Refined final search terms for monthly updates.

Additional file 2. References of initial library (*n* = 102).

## Data Availability

The search strings are located in the supplementary. The library of studies is currently not publicly available but plans are underway to make it available.

## Abbreviations

ALEC: Australian Living Evidence Collaboration
HIQA: Health Information and Quality Authority
RCT: Randomised controlled trial
SR: Systematic review
PASC: Post-acute sequelae of COVID-19
