# Supplementary material for: Living evidence syntheses for long COVID therapeutics: combining rigorous protocols to build efficiency while maintaining rigour
Source: Syst Rev. 2026 Apr 2;15:165. doi: 10.1186/s13643-026-03178-x (PMC13169727; doi:10.1186/s13643-026-03178-x)
Supplement: Supplementary file 2 — Additional file 2. References of initial library (n = 102). [file 13643_2026_3178_MOESM2_ESM.docx]

**Additional File 2.** References of initial library (n=102)

1. Abdelazim MH, Abdelazim AH. Effect of Sodium Gluconate on Decreasing Elevated Nasal Calcium and Improving Olfactory Function Post COVID-19 Infection. Am J Rhinol Allergy. 2022;36(6):841-8.

2. Abdelazim MH, Abdelazim AH, Moneir W. The effect of intra-nasal tetra sodium pyrophosphate on decreasing elevated nasal calcium and improving olfactory function post COVID-19: a randomized controlled trial. Allergy Asthma Clin Immunol. 2022;18(1):67.

3. Abdelazim MH, Mandour Z, Abdelazim AH, Ismaiel WF, Gamal M, Abourehab MAS, et al. Intra Nasal Use of Ethylene Diamine Tetra Acetic Acid for Improving Olfactory Dysfunction Post COVID-19. Am J Rhinol Allergy. 2023;37(6):630-7.

4. Abo El Naga H, El Zaiat R, Hamdan A. The potential therapeutic effect of platelet-rich plasma in the treatment of post-COVID-19 parosmia. The Egyptian Journal of Otolaryngology. 2022;38.

5. Abo Elyazed TI, Abd El-Hakim AAE, Saleh OI, Sonbol MMF, Eid HA, Moazen E, et al. Diaphragmatic strengthening exercises for patients with post COVID-19 condition after mild-to-moderate acute COVID-19 infection: a randomized controlled study. J Rehabil Med. 2024;56:jrm25491.

6. Ahmad AM, Mohamed Awad Allah SA, Abd Elhaseeb GA, Elsharawy DE, Ahmed HS, Mohamed Abdelwahab MA. Effects of conventional versus virtual reality-simulated treadmill exercise on fatigue, cognitive function, and participant satisfaction in post-COVID-19 subjects. A randomized trial. J Exerc Sci Fit. 2024;22(4):316-21.

7. Alsharidah A, Kamel F, Alanazi A, Alhawsah E, Alharbi H, Alrshedi Z, et al. A Pulmonary Telerehabilitation Program Improves Exercise Capacity and Quality of Life in Young Females Post-COVID-19 Patients. Annals of rehabilitation medicine. 2023;47:502-10.

8. Altemani A, Alanazi M, Altemani A, Alharbi A, Alsahali S, Alotaib N, et al. The Efficacy of Sodium Phytate as a Natural Chelating Agent in Reducing Elevated Calcium Levels in Nasal Mucus Among Individuals Experiencing Olfactory Dysfunction Following COVID-19: A Prospective Randomized Double-Controlled Clinical Trial. American journal of rhinology & allergy. 2023;38:116-22.

9. Amorim NTS, Cavalcanti FCB, Moura E, Sobral Filho D, Leitão CCS, Almeida MM, et al. Does whole-body vibration improve risk of falls, balance, and heart rate variability in post-COVID-19 patients? A randomized clinical trial. J Bodyw Mov Ther. 2024;39:518-24.

10. Andrews JS, Boonyaratanakornkit JB, Krusinska E, Allen S, Posada JA. Assessment of the Impact of RNase in Patients With Severe Fatigue Related to Post-Acute Sequelae of SARS-CoV-2 Infection (PASC): A Randomized Phase 2 Trial of RSLV-132. Clin Infect Dis. 2024.

11. Badran B, Huffman S, Dancy M, Austelle C, Bikson M, Kautz S, et al. A pilot randomized controlled trial of supervised, at-home, self-administered transcutaneous auricular vagus nerve stimulation (taVNS) to manage long COVID symptoms. Bioelectronic medicine. 2022;8:13.

12. Berenguel Senén A, Gadella Fernández A, Godoy López J, Borrego Rodríguez J, Gallango Brejano M, Cepas Guillén P, et al. Functional rehabilitation based on therapeutic exercise training in patients with postacute COVID syndrome (RECOVER). Revista espanola de cardiologia (English ed). 2023;77:167-75.

13. Bérubé S, Demers C, Bussière N, Cloutier F, Pek V, Chen A, et al. Olfactory Training Impacts Olfactory Dysfunction Induced by COVID-19: A Pilot Study. ORL J Otorhinolaryngol Relat Spec. 2023;85(2):57-66.

14. Besnier F, Malo J, Mohammadi H, Clavet S, Klai C, Martin N, et al. Effects of Cardiopulmonary Rehabilitation on Cardiorespiratory Fitness and Clinical Symptom Burden in Long COVID: Results from the COVID-Rehab Randomized Controlled Trial. Am J Phys Med Rehabil. 2024.

15. Bhandari R. Online Yoga and Ayurveda Intervention as Tertiary Prevention of Psychological Comorbidities in COVID-19 Survivors: A Randomized Controlled Trial. Annals of neurosciences. 2022;29:233-44.

16. Bileviciute-Ljungar I, Norrefalk J, Borg K. Improved Functioning and Activity According to the International Classification of Functioning and Disability after Multidisciplinary Telerehabilitation for Post-COVID-19 Condition-A Randomized Control Study. Journal of clinical medicine. 2024;13:970-.

17. Bowen R, Arany P. Use of either transcranial or whole-body photobiomodulation treatments improves COVID-19 brain fog. Journal of biophotonics. 2023;16:e202200391.

18. Brichetti V, Rubilar T, Tejada JV, Montecino P, Crespi-Abril AC, Barbieri ES, et al. EuroQol-5D-3L in Long Covid patients After Supplementation with EchA Marine, a Sea Urchin Eggs Extract: a double-blinded, multicentrical study. medRxiv. 2023.

19. Calvo-Paniagua J, Díaz-Arribas MJ, Valera-Calero JA, Ramos-Sánchez M, Fernández-de-Las-Peñas C, Navarro-Santana MJ, et al. An Educational, Exercise and Occupational Therapy-Based Telerehabilitation Program versus 'Wait-and-See' for Improving Self-Perceived Exertion in Patients with post-COVID Fatigue and Dyspnea: A Randomized Clinical Trial. Am J Phys Med Rehabil. 2024.

20. Cantone E, D'Ascanio L, De Luca P, Roccamatisi D, La La Mantia I, Brenner M, et al. Persistent COVID-19 parosmia and olfactory loss post olfactory training: randomized clinical trial comparing central and peripheral-acting therapeutics. European archives of oto-rhino-laryngology : official journal of the European Federation of Oto-Rhino-Laryngological Societies (EUFOS) : affiliated with the German Society for Oto-Rhino-Laryngology - Head and Neck Surgery. 2024.

21. Cardoso Soares P, de Freitas P, Eduardo C, Azevedo L. Photobiomodulation, Transmucosal Laser Irradiation of Blood, or B complex as alternatives to treat Covid-19 Related Long-Term Taste Impairment: double-blind randomized clinical trial. Lasers in medical science. 2023;38:261.

22. Catalogna M, Sasson E, Hadanny A, Parag Y, Zilberman-Itskovich S, Efrati S. Effects of hyperbaric oxygen therapy on functional and structural connectivity in post-COVID-19 condition patients: A randomized, sham-controlled trial. NeuroImage Clinical. 2022;36:103218-.

23. Çelik Z, Kafa N, Güzel NA, Köktürk N. The effects of physical activity tele-counseling intervention on physical activity, functional performance, and quality of life in post-COVID-19 conditions: a randomized controlled trial. Expert Rev Respir Med. 2024.

24. Chung T, Zhang H, Wong F, Sridhar S, Lee T, Leung G, et al. A Pilot Study of Short-Course Oral Vitamin A and Aerosolised Diffuser Olfactory Training for the Treatment of Smell Loss in Long COVID. Brain sciences. 2023;13:1014-.

25. D'Ascanio L, Vitelli F, Cingolani C, Maranzano M, Brenner MJ, Di Stadio A. Randomized clinical trial "olfactory dysfunction after COVID-19: olfactory rehabilitation therapy vs. intervention treatment with Palmitoylethanolamide and Luteolin": preliminary results. Eur Rev Med Pharmacol Sci. 2021;25(11):4156-62.

26. Dal Negro R, Turco P, Povero M. Nebivolol: an effective option against long-lasting dyspnoea following COVID-19 pneumonia - a pivotal double-blind, cross-over controlled study. Multidisciplinary respiratory medicine. 2022;17:886.

27. De Luca P, Camaioni A, Marra P, Salzano G, Carriere G, Ricciardi L, et al. Effect of Ultra-Micronized Palmitoylethanolamide and Luteolin on Olfaction and Memory in Patients with Long COVID: Results of a Longitudinal Study. Cells. 2022;11(16):2552-.

28. Del Corral T, Fabero-Garrido R, Plaza-Manzano G, Fernández-de-Las-Peñas C, Navarro-Santana M, López-de-Uralde-Villanueva I. Home-based respiratory muscle training on quality of life and exercise tolerance in long-term post-COVID-19: Randomized controlled trial. Ann Phys Rehabil Med. 2023;66(1):101709.

29. Di Stadio A, Cantone E, De Luca P, Di Nola C, Massimilla E, Motta G, et al. Parosmia COVID-19 Related Treated by a Combination of Olfactory Training and Ultramicronized PEA-LUT: A Prospective Randomized Controlled Trial. Biomedicines. 2023;11:1109-.

30. Di Stadio A, D'Ascanio L, Vaira LA, Cantone E, De Luca P, Cingolani C, et al. Ultramicronized Palmitoylethanolamide and Luteolin Supplement Combined with Olfactory Training to Treat Post-COVID-19 Olfactory Impairment: A Multi-Center Double-Blinded Randomized Placebo- Controlled Clinical Trial. Curr Neuropharmacol. 2022;20(10):2001-12.

31. Di Stadio A, Gallina S, Cocuzza S, De Luca P, Ingrassia A, Oliva S, et al. Treatment of COVID-19 olfactory dysfunction with olfactory training, palmitoylethanolamide with luteolin, or combined therapy: a blinded controlled multicenter randomized trial. European archives of oto-rhino-laryngology : official journal of the European Federation of Oto-Rhino-Laryngological Societies (EUFOS) : affiliated with the German Society for Oto-Rhino-Laryngology - Head and Neck Surgery. 2023;280:4949-61.

32. Duffy A, Naimi B, Garvey E, Hunter S, Kumar A, Kahn C, et al. Topical platelet‐rich plasma as a possible treatment for olfactory dysfunction—A randomized controlled trial. International Forum of Allergy & Rhinology. 2024.

33. Espinoza-Bravo C, Arnal-Gómez A, Martínez-Arnau FM, Núñez-Cortés R, Hernández-Guillén D, Flor-Rufino C, et al. Effectiveness of Functional or Aerobic Exercise Combined With Breathing Techniques in Telerehabilitation for Patients With Long COVID: A Randomized Controlled Trial. Phys Ther. 2023;103(11).

34. Evman M, Cetin Z. Effectiveness of platelet-rich plasma on post-COVID chronic olfactory dysfunction. Revista da Associacao Medica Brasileira (1992). 2023;69:e20230666.

35. Figueiredo L, Paim P, Cerqueira-Silva T, Barreto C, Lessa M. Alpha-lipoic acid does not improve olfactory training results in olfactory loss due to COVID-19: a double-blind randomized trial. Brazilian journal of otorhinolaryngology. 2023;90:101356-.

36. Finnigan LEM, Cassar MP, Koziel MJ, Pradines J, Lamlum H, Azer K, et al. Efficacy and tolerability of an endogenous metabolic modulator (AXA1125) in fatigue-predominant long COVID: a single-centre, double-blind, randomised controlled phase 2a pilot study. EClinicalMedicine. 2023;59:101946.

37. Gaudreau-Majeau F, Gagnon C, Djedaa S, Bérubé B, Malo J, Iglesies-Grau J, et al. Cardiopulmonary rehabilitation's influence on cognitive functions, psychological state, and sleep quality in long COVID-19 patients: A randomized controlled trial. Neuropsychological rehabilitation. 2024:1-17.

38. Gaylis NB, Ritter A, Kelly SA, Pourhassan NZ, Tiwary M, Sacha JB, et al. Reduced Cell Surface Levels of C-C Chemokine Receptor 5 and Immunosuppression in Long Coronavirus Disease 2019 Syndrome. Clin Infect Dis. 2022;75(7):1232-4.

39. Gomes Dos Santos EG, Vieira da Costa K, Cordeiro de Souza IT, Victor Dos Santos Felix J, Furtado Brandão CB, Michelle de Souza Fernandes V, et al. Effects of a cardiopulmonary rehabilitation protocol on functional capacity, dyspnea, fatigue, and body composition in individuals with post-COVID-19 syndrome: A randomized controlled trial. Physiother Res Int. 2024;29(2):e2086.

40. González-Moreno J, Pozuelo C, Manos D, Gómez-Martínez S, Cantero-García M. A third generation therapies approach in long covid patients: Efficacy of an intervention program with spanish adults. Current Psychology: A Journal for Diverse Perspectives on Diverse Psychological Issues. 2024.

41. Gupta S, Lee JJ, Perrin A, Khan A, Smith HJ, Farrell N, et al. Efficacy and Safety of Saline Nasal Irrigation Plus Theophylline for Treatment of COVID-19-Related Olfactory Dysfunction: The SCENT2 Phase 2 Randomized Clinical Trial. JAMA Otolaryngol Head Neck Surg. 2022;148(9):830-7.

42. Hamed S, Ahmed M. The effectiveness of cerebrolysin, a multi-modal neurotrophic factor, for treatment of post-covid-19 persistent olfactory, gustatory and trigeminal chemosensory dysfunctions: a randomized clinical trial. Expert review of clinical pharmacology. 2023;16:1261-76.

43. Hansen KS, Mogensen TH, Agergaard J, Schiøttz-Christensen B, Østergaard L, Vibholm LK, et al. High-dose coenzyme Q10 therapy versus placebo in patients with post COVID-19 condition: a randomized, phase 2, crossover trial. Lancet Reg Health Eur. 2023;24:100539.

44. Hawkins J, Hires C, Keenan L, Dunne E. Aromatherapy blend of thyme, orange, clove bud, and frankincense boosts energy levels in post-COVID-19 female patients: A randomized, double-blinded, placebo controlled clinical trial. Complement Ther Med. 2022;67:102823.

45. Hintschich CA, Dietz M, Haehner A, Hummel T. Topical Administration of Mometasone Is Not Helpful in Post-COVID-19 Olfactory Dysfunction. Life (Basel). 2022;12(10).

46. Imam MS, Abdelazim MH, Abdelazim AH, Ismaiel WF, Gamal M, Abourehab MAS, et al. Efficacy of pentasodium diethylenetriamine pentaacetate in ameliorating anosmia post COVID-19. Am J Otolaryngol. 2023;44(4):103871.

47. Jimeno-Almazán A, Buendía-Romero Á, Martínez-Cava A, Franco-López F, Sánchez-Alcaraz BJ, Courel-Ibáñez J, et al. Effects of a concurrent training, respiratory muscle exercise, and self-management recommendations on recovery from post-COVID-19 conditions: the RECOVE trial. J Appl Physiol (1985). 2023;134(1):95-104.

48. Jimeno-Almazán A, Franco-López F, Buendía-Romero Á, Martínez-Cava A, Sánchez-Agar JA, Sánchez-Alcaraz Martínez BJ, et al. Rehabilitation for post-COVID-19 condition through a supervised exercise intervention: A randomized controlled trial. Scand J Med Sci Sports. 2022;32(12):1791-801.

49. Kaczmarczyk K, Matharu Y, Bobowik P, Gajewski J, Maciejewska-Skrendo A, Kulig K. Resistance Exercise Program Is Feasible and Effective in Improving Functional Strength in Post-COVID Survivors. Journal of clinical medicine. 2024;13:1712-.

50. Kerget B, Çil G, Araz Ö, Alper F, Akgün M. Comparison of two antifibrotic treatments for lung fibrosis in post-COVID-19 syndrome: A randomized, prospective study. Medicina clinica (English ed). 2023;160:525-30.

51. Kerling A, Beyer S, Dirks M, Scharbau M, Hennemann A, Dopfer-Jablonka A, et al. Effects of a randomized-controlled and online-supported physical activity intervention on exercise capacity, fatigue and health related quality of life in patients with post-COVID-19 syndrome. BMC sports science, medicine & rehabilitation. 2024;16:33.

52. Khan AM, Piccirillo J, Kallogjeri D, Piccirillo JF. Efficacy of Combined Visual-Olfactory Training With Patient-Preferred Scents as Treatment for Patients With COVID-19 Resultant Olfactory Loss: A Randomized Clinical Trial. JAMA Otolaryngol Head Neck Surg. 2023;149(2):141-9.

53. Klírová M, Adamová A, Biačková N, Laskov O, Renková V, Stuchlíková Z, et al. Transcranial direct current stimulation (tDCS) in the treatment of neuropsychiatric symptoms of long COVID. Sci Rep. 2024;14(1):2193.

54. Kogel A, Machatschek M, Scharschmidt R, Wollny C, Lordick F, Ghanem M, et al. Physical exercise as a treatment for persisting symptoms post-COVID infection: review of ongoing studies and prospective randomized controlled training study. Clin Res Cardiol. 2023;112(11):1699-709.

55. Kuut T, Müller F, Csorba I, Braamse A, Aldenkamp A, Appelman B, et al. Efficacy of Cognitive-Behavioral Therapy Targeting Severe Fatigue Following Coronavirus Disease 2019: Results of a Randomized Controlled Trial. Clinical infectious diseases : an official publication of the Infectious Diseases Society of America. 2023;77:687-95.

56. Kwan ATH, Guo Z, Ceban F, Le GH, Wong S, Teopiz KM, et al. Assessing the Effects of Metabolic Disruption, Body Mass Index and Inflammation on Depressive Symptoms in Post-COVID-19 Condition: A Randomized Controlled Trial on Vortioxetine. Adv Ther. 2024;41(5):1983-94.

57. Lai CY, Lin CH, Chao TC, Chang CC, Huang CY, Chiang SL. Effectiveness of a 12-week telerehabilitation training in people with long COVID: A randomized controlled trial. Ann Phys Rehabil Med. 2024;67(5):101853.

58. Lasheen H, Abou-Zeid M. Olfactory mucosa steroid injection in treatment of post-COVID-19 olfactory dysfunction: a randomized control trial. The Egyptian Journal of Otolaryngology. 2023;39.

59. Lau RI, Su Q, Lau ISF, Ching JYL, Wong MCS, Lau LHS, et al. A synbiotic preparation (SIM01) for post-acute COVID-19 syndrome in Hong Kong (RECOVERY): a randomised, double-blind, placebo-controlled trial. Lancet Infect Dis. 2024;24(3):256-65.

60. Leitman M, Fuchs S, Tyomkin V, Hadanny A, Zilberman-Itskovich S, Efrati S. The effect of hyperbaric oxygen therapy on myocardial function in post-COVID-19 syndrome patients: a randomized controlled trial. Scientific reports. 2023;13:9473.

61. Lerner D, Garvey K, Arrighi-Allisan A, Kominsky E, Filimonov A, Al-Awady A, et al. Omega-3 Fatty Acid Supplementation for the Treatment of Persistent COVID-Related Olfactory Dysfunction. American journal of rhinology & allergy. 2023;37:531-40.

62. M K, A B, L D, P G, B D, P dT, et al. Feasibility of a Group-Based Telerehabilitation Intervention for Long COVID Management. ResearchSquare. 2022.

63. Mahadev A, Hentati F, Miller B, Bao J, Perrin A, Kallogjeri D, et al. Efficacy of Gabapentin For Post-COVID-19 Olfactory Dysfunction: The GRACE Randomized Clinical Trial. JAMA otolaryngology-- head & neck surgery. 2023;149:1111-.

64. Marinoni B, Rimondi A, Bottaro F, Ciafardini C, Amoroso C, Muià M, et al. The Role of VSL#3® in the Treatment of Fatigue and Other Symptoms in Long Covid-19 Syndrome: a Randomized, Double-blind, Placebo-controlled Pilot Study (DELong#3). 2023.

65. McGregor G, Sandhu H, Bruce J, Sheehan B, McWilliams D, Yeung J, et al. Clinical effectiveness of an online supervised group physical and mental health rehabilitation programme for adults with post-covid-19 condition (REGAIN study): multicentre randomised controlled trial. Bmj. 2024;384:e076506.

66. McIntyre RS, Phan L, Kwan ATH, Mansur RB, Rosenblat JD, Guo Z, et al. Vortioxetine for the treatment of post-COVID-19 condition: a randomized controlled trial. Brain. 2024;147(3):849-57.

67. McNarry MA, Berg RMG, Shelley J, Hudson J, Saynor ZL, Duckers J, et al. Inspiratory muscle training enhances recovery post-COVID-19: a randomised controlled trial. Eur Respir J. 2022;60(4).

68. Mooren J, Garbsch R, Schäfer H, Kotewitsch M, Waranski M, Teschler M, et al. Medical Rehabilitation of Patients with Post-COVID-19 Syndrome-A Comparison of Aerobic Interval and Continuous Training. Journal of clinical medicine. 2023;12:6739-.

69. Navas-Otero A, Calvache-Mateo A, Calles-Plata I, Valenza-Peña G, Hernández-Hernández S, Ortiz-Rubio A, et al. A lifestyle adjustments program in long COVID-19 improves symptomatic severity and quality of life. A randomized control trial. Patient Educ Couns. 2024;122:108180.

70. Okan F, Okan S, Duran Yücesoy F. Evaluating the Efficiency of Breathing Exercises via Telemedicine in Post-Covid-19 Patients: Randomized Controlled Study. Clin Nurs Res. 2022;31(5):771-81.

71. Oliver-Mas S, Delgado-Alonso C, Delgado-Álvarez A, Díez-Cirarda M, Cuevas C, Fernández-Romero L, et al. Transcranial direct current stimulation for post-COVID fatigue: a randomized, double-blind, controlled pilot study. Brain Commun. 2023;5(2):fcad117.

72. Orlova EV, Lyamina NP, Skorobogatyth NV, Pogonchenkova IV. Сlinical Efficacy of Individually Dosed Intermittent Hypoxia-Hyperoxic Therapy in Osteoarthritis Patients with Post-Covid Syndrome. Bulletin of Rehabilitation Medicine. 2022;21(2):6-16.

73. Palau P, Domínguez E, Gonzalez C, Bondía E, Albiach C, Sastre C, et al. Effect of a home-based inspiratory muscle training programme on functional capacity in postdischarged patients with long COVID: the InsCOVID trial. BMJ Open Respir Res. 2022;9(1).

74. Philip KEJ, Owles H, McVey S, Pagnuco T, Bruce K, Brunjes H, et al. An online breathing and wellbeing programme (ENO Breathe) for people with persistent symptoms following COVID-19: a parallel-group, single-blind, randomised controlled trial. Lancet Respir Med. 2022;10(9):851-62.

75. Pietranis KA, Izdebska WM, Kuryliszyn-Moskal A, Dakowicz A, Ciołkiewicz M, Kaniewska K, et al. Effects of Pulmonary Rehabilitation on Respiratory Function and Thickness of the Diaphragm in Patients with Post-COVID-19 Syndrome: A Randomized Clinical Trial. J Clin Med. 2024;13(2).

76. Pleguezuelos E, Del Carmen A, Moreno E, Miravitlles M, Serra M, Garnacho-Castaño M. Effects of a telerehabilitation program and detraining on cardiorespiratory fitness in patients with post-COVID-19 sequelae: A randomized controlled trial. Scandinavian journal of medicine & science in sports. 2023;34:e14543.

77. Pleguezuelos E, Del Carmen A, Moreno E, Serra-Prat M, Serra-Payá N, Garnacho-Castaño MV. Telerehabilitation improves cardiorespiratory and muscular fitness and body composition in older people with post-COVID-19 syndrome. J Cachexia Sarcopenia Muscle. 2024.

78. Romanet C, Wormser J, Fels A, Lucas P, Prudat C, Sacco E, et al. Effectiveness of exercise training on the dyspnoea of individuals with long COVID: A randomised controlled multicentre trial. Ann Phys Rehabil Med. 2023;66(5):101765.

79. Saha S, Singh R, Mani I, Chakraborty K, Sarkar P, Rana A, et al. Individualized Homeopathic Medicines in the Treatment of Post-COVID-19 Fatigue in Adults: Single-Blind, Randomized, Placebo-Controlled Trial. Complement Med Res. 2024;31(1):1-9.

80. Samper-Pardo M, León-Herrera S, Oliván-Blázquez B, Méndez-López F, Domínguez-García M, Sánchez-Recio R. Effectiveness of a telerehabilitation intervention using ReCOVery APP of long COVID patients: a randomized, 3-month follow-up clinical trial. Scientific reports. 2023;13:7943.

81. Samper-Pardo M, Oliván-Blázquez B, León-Herrera S, Sánchez-Arizcuren R, Casado-Vicente V, Sánchez-Recio R. Effectiveness of ReCOVery APP to improve the quality of life of Long COVID patients: a 6-month follow-up randomized clinical trial. 2023.

82. Sánchez Milá Z, Rodríguez Sanz D, Martín Nieto A, Jiménez Lobo A, Ramos Hernández M, Campón Chekroun A, et al. Effects of a respiratory and neurological rehabilitation treatment plan in post Covid-19 affected university students. Randomized clinical study. Chronic Respiratory Disease. 2024;21.

83. Sánchez-Milá Z, Abuín-Porras V, Romero-Morales C, Almazán-Polo J, Velázquez Saornil J. Effectiveness of a respiratory rehabilitation program including an inspiration training device versus traditional respiratory rehabilitation: a randomized controlled trial. PeerJ. 2023;11:e16360-e.

84. Santana K, França E, Sato J, Silva A, Queiroz M, de Farias J, et al. Non-invasive brain stimulation for fatigue in post-acute sequelae of SARS-CoV-2 (PASC). Brain Stimul. 2023;16(1):100-7.

85. Sarmento A, Adodo R, Hodges G, Webber S, Sanchez-Ramirez D. Virtual pulmonary rehabilitation approaches in patients with post COVID syndrome: a pilot study. BMC pulmonary medicine. 2024;24:139.

86. Schmidt F, Azar C, Goektas O. Treatment of Olfactory Disorders After SARS - CoViD 2 Virus Infection. Ear, nose, & throat journal. 2023:1455613231168487-014556132311684.

87. Shatri H, Sinulingga DI, Rumende CM, Setiati S, Putranto R, Ginanjar E, et al. Effectiveness of Internet-Based Group Supportive Psychotherapy on Psychic and Somatic Symptoms, Neutrophil-Lymphocyte Ratio, and Heart Rate Variability in Post COVID-19 Syndrome Patients. Acta Med Indones. 2023;55(4):411-20.

88. Shogenova LV, Truong TT, Kryukova NO, Yusupkhodzhaeva KA, Pozdnyakova DD, Kim TG, et al. Hydrogen inhalation in rehabilitation program of the medical staff recovered from COVID-19. Cardiovascular Therapy and Prevention. 2021;20(6).

89. Slankamenac J, Ranisavljev M, Todorovic N, Ostojic J, Stajer V, Ostojic SM. Creatine supplementation combined with breathing exercises reduces respiratory discomfort and improves creatine status in patients with long-COVID. J Postgrad Med. 2024;70(2):101-4.

90. Spiesshoefer J, Regmi B, Senol M, Jörn B, Gorol O, Elfeturi M, et al. Potential Diaphragm Muscle Weakness-related Dyspnea Persists Two Years after COVID-19 and Could Be Improved by Inspiratory Muscle Training: Results of an Observational and an Interventional Trial. Am J Respir Crit Care Med. 2024.

91. Stölting A, Schröder D, Müllenmeister C, Behrens GMN, Klawitter S, Klawonn F, et al. Improvement in quality of life and cognitive function in Post Covid Syndrome after online occupational therapy: results from a randomized controlled pilot study. medRxiv. 2024.

92. Sumbalová Z, Kucharská J, Rausová Z, Palacka P, Kovalčíková E, Takácsová T, et al. Reduced platelet mitochondrial respiration and oxidative phosphorylation in patients with post COVID-19 syndrome are regenerated after spa rehabilitation and targeted ubiquinol therapy. Front Mol Biosci. 2022;9:1016352.

93. Tanashyan M, Morozova S, Raskurazhev A, Kuznetsova P. A prospective randomized, double-blind placebo-controlled study to evaluate the effectiveness of neuroprotective therapy using functional brain MRI in patients with post-covid chronic fatigue syndrome. Biomed Pharmacother. 2023;168:115723.

94. Tanashyan MM, Raskurazhev AA, Kuznetsova PI, Bely PA, Zaslavskaya KI. [Prospects and possibilities for the treatment of patients with long COVID-19 syndrome]. Ter Arkh. 2022;94(11):1285-93.

95. Tosato M, Calvani R, Picca A, Ciciarello F, Galluzzo V, Coelho-Júnior HJ, et al. Effects of l-Arginine Plus Vitamin C Supplementation on Physical Performance, Endothelial Function, and Persistent Fatigue in Adults with Long COVID: A Single-Blind Randomized Controlled Trial. Nutrients. 2022;14(23).

96. Toussaint LL, Bratty AJ. Amygdala and Insula Retraining (AIR) Significantly Reduces Fatigue and Increases Energy in People with Long COVID. Evid Based Complement Alternat Med. 2023;2023:7068326.

97. Tryfonos A, Pourhamidi K, Jörnåker G, Engvall M, Eriksson L, Elhallos S, et al. Functional Limitations and Exercise Intolerance in Patients With Post-COVID Condition: A Randomized Crossover Clinical Trial. JAMA Netw Open. 2024;7(4):e244386.

98. Vallier JM, Simon C, Bronstein A, Dumont M, Jobic A, Paleiron N, et al. Randomized controlled trial of home-based vs. hospital-based pulmonary rehabilitation in post COVID-19 patients. Eur J Phys Rehabil Med. 2023;59(1):103-10.

99. Versace V, Ortelli P, Dezi S, Ferrazzoli D, Alibardi A, Bonini I, et al. Co-ultramicronized palmitoylethanolamide/luteolin normalizes GABA(B)-ergic activity and cortical plasticity in long COVID-19 syndrome. Clin Neurophysiol. 2023;145:81-8.

100. Yan CH, Jang SS, Lin HC, Ma Y, Khanwalkar AR, Thai A, et al. Use of platelet-rich plasma for COVID-19-related olfactory loss: a randomized controlled trial. Int Forum Allergy Rhinol. 2023;13(6):989-97.

101. Zilberman-Itskovich S, Catalogna M, Sasson E, Elman-Shina K, Hadanny A, Lang E, et al. Hyperbaric oxygen therapy improves neurocognitive functions and symptoms of post-COVID condition: randomized controlled trial. Sci Rep. 2022;12(1):11252.

102. Zulbaran-Rojas A, Lee M, Bara R, Flores-Camargo A, Spitz G, Finco M, et al. Electrical stimulation to regain lower extremity muscle perfusion and endurance in patients with post-acute sequelae of SARS CoV-2: A randomized controlled trial. Physiological reports. 2023;11:e15636.
